# Determining the readiness of patients with renal failure to use health information technology

**DOI:** 10.1186/s12911-022-02073-4

**Published:** 2022-12-08

**Authors:** Raana Younesi Tabas, Leila Ahmadian, Mahnaz Samadbeik, Anahita Arian, Arefeh Ameri

**Affiliations:** 1 Health Information Management Department, Valiasr Hospital, Birjand University of Medical Sciences, Birjand, Iran; 2 Health Information Sciences Department, Faculty of Management and Medical Information Sciences, Kerman University of Medical Sciences, Kerman, Iran; 3 Social Determinants of Health Research Center, Lorestan University of Medical Sciences, Lorestan, Iran; 4 Department of Internal Medicine, Cardiovascular Diseases Research Center Valiasr Hospital, Birjand University of Medical Sciences, Birjand, Iran; 5 Health Information Sciences Department, Faculty of Management and Medical Information Sciences, Kerman University of Medical Sciences, Kerman, Iran

**Keywords:** Kidney failure, Health information technology, Patient preparation, Use of the internet

## Abstract

**Introduction:**

Using information technology (IT) for purposes such as patient education and disease prevention and management is effective when patients are ready to use it. The objective of this study was to determine the readiness of patients with renal failure to use health IT.

**Methods:**

This study was performed on all dialysis patients in South Khorasan province (n = 263) using a 28-item questionnaire. The questionnaire consisted of (1) demographic information of participants and (2) questions concerning eight main factors including the need for information, desire to receive information, ability to use computers and the Internet, computers and the Internet anxiety, communication with physicians, using mobile phones and concerns about security and confidentiality of information. Descriptive statistics and Mann–Whitney and Kruskal–Wallis statistical tests were used to analyze the data.

**Results:**

About 15% of the participants stated that they do not want to receive information from the Internet. Anxiety and concern about Internet security and confidentiality were higher in women, married people, people over 60, villagers, and illiterate people (*p* < 0.05). Married people and people over 60 years had a higher desire to get information (*p* < 0.05). The rate of computer anxiety and Internet privacy concern was higher than average (*p* < 0.001). Most patients (34.2%) could only send text messages using mobile phones.

**Conclusion:**

Despite the need of most patients to online health information, they do not use this information due to a lack of skills and experience to use IT. Therefore, the ability of users should be considered when developing IT-based interventions. Due to patients' concerns about Internet privacy, it is required to teach patients how to protect their privacy while using the Internet.

## Introduction

Chronic kidney disease is caused by the progressive and irreversible destruction of the nephrons. In such a case, the body's ability to maintain metabolism and balance water and electrolytes is lost, resulting in increased blood urea. The population of patients with chronic kidney disease mostly includes older adults and people with lower social and health literacy status [1].

At the end of 2016, the number of dialysis patients is estimated 2989,000 million worldwide. In general, more than 50% of dialysis patients live in five countries: the United States (US), China, Japan, Brazil, and India. The US Renal Data System Annual Data Report showed that the prevalence of chronic kidney disease in adults during the period 2014–2011 was 14.8%. The cost of US medical insurance for the elderly for chronic kidney disease patients aged 65 and over in 2014 was more than $ 50 billion, representing 20% ​​of the total cost of US medical insurance for the elderly in this age group. According to the American Society of Nephrology and the Center for Disease Control and Prevention (CDC), the disease has increased by 30% in the last decade [2]. Iran has also faced a growing trend of dialysis patients, so that by the end of 2016 the population of patients with end-stage renal disease, who were treated by one of the kidney replacement methods, reached about 58,000 patients [3]. Many of these patients are elderly. In previous years, the main goal of medical care was to increase the survival rate, but in recent years, this goal has been to improve the quality of life for patients [4]. In Iran, more than 14 million people have chronic kidney disease, and it increases by 15–17% every year. Most patients only see a doctor when their disease has progressed and they need dialysis or a kidney transplant, which is expensive [5]. Untreated chronic kidney disease not only increases the risk of developing end-stage renal disease but also increases the risk of cardiovascular mortality [6].

Proper management of health services and empowering the community for self-care help to prevent these diseases and avoid the deterioration of patients’ conditions [7]. Early diagnosis and accurate prognosis of these diseases using risk prediction models facilitate the initiation of appropriate treatment, early monitoring of patients' conditions, and referral to specialists, leading to improvement of outcomes [8]. Low economic status and inadequate health literacy of patients reduce patient participation in decision-making about their treatment, lower access to preventive services, weaken the control of chronic diseases, and ultimately increases the use of emergency units and hospital services by these patients [9]. Currently, hospitals and health systems rely on information and communication technology as a means to improve the quality, safety, and efficiency of medical services [10]. The impact of IT on the management of kidney disease has been identified, and it has suggested several ways to manage patients with chronic kidney disease [11]. The use of a clinical decision support system can improve the quality of decisions made by physicians when prescribing medication to patients with renal failure [12]. With the advances of online and mobile health interventions for changing lifestyle and self-management of chronic disease, self-management using interactive online interventions is becoming one of the important components that support patients in better self-management of chronic diseases [13].

The results of a study showed that the best ways to prevent chronic diseases are managing healthcare services properly and enabling people to use new information and communication technology tools [7]. The use of open-source electronic health records has improved diabetes management, control of hypertension, and increased TB vaccination [14]. Individuals can contact a family physician, specialist physician, or other health professionals via email and the web to request a medication order or refill, make an appointment and ask specific health questions [15]. The satisfaction of users with electronic messaging as a communication channel between patients and physicians was revealed by a study. The results showed that patients tend to use these services due to their convenience, time savings, and economic benefits [16].

Due to the increasing number of chronic kidney patients and the occurrence of cardiovascular complications, renal failure, and subsequent increases in treatment costs, disability, and death, the development of educational programs to reduce these problems seems necessary. The widespread use and availability of IT to most people, and its potential to provide information, makes it an effective intervention for developing such programs. Therefore, the objective of this study was to determine the readiness of chronic kidney patients under hemodialysis in South Khorasan, IRAN to use health information technology. High number of chronic kidney patients live in this region and a range of 20–44 presents of these patients are undergoing long-term dialysis therapy. The results can help policymakers to plan and develop IT-based training and self-care applications to control deaths from chronic kidney disease.

## Methods

### Study population

This survey study was conducted on all chronic kidney patients who were admitted for dialysis at one of the nine dialysis units of Birjand University of Medical Sciences, Social Security Hospital of South Khorasan Province, and a dialysis referral center in Birjand. The study was done on all patients (n = 263) who visited these health centers during six month (2019). Patients who diagnosed with end-stage chronic kidney disease and received hemodialysis were included in this study.

### Data collection tool

In this study, the questionnaire "Patient readiness to engage in health information technology (PRE-HIT)" developed by Koopman et al. was used [13]. This questionnaire was first translated into Persian and experts in the domain of medical sciences and English language and literature approved its translation. Then, to check the cross-cultural reliability, the Persian version of the questionnaire was translated into English and was compared with the original version by a bilingual expert. The face validity of this final questionnaire was confirmed by three specialists in the field of medical informatics and seven nursing faculty members. In the study of Koopman et al., The reliability of the English version of this questionnaire was 0.95 [13]. The test–retest method was used to assess the reliability of the Persian version. For this purpose, the final Persian questionnaire was given to 21 dialysis patients in Birjand two weeks apart. The retest coefficient and Cronbach's alpha in this study were 0.96 and 0.79, respectively.

The questionnaire consisted of two parts. The first part was designed to collect demographic information of the participants (age, sex, education level, marital status, duration of illness). The second part included questions for assessing patients' readiness to use health technology. This part of the questionnaire covered the following eight main factors (including 28 items); health information need (5 items), desire to receive information (3 items), computer/internet experience and expertise (4 items), computer/internet anxiety (4 items), preferred mode of interaction (5 items), relationship with doctor (with 3 items), cell phone expertise (2 items), and internet privacy concerns (2 items). The answers to the questions could be provided on a four-point Likert scale from "Completely Agree" to "Completely Disagree".

### Data collection method

One of the researchers distributed the questionnaire among participants in the dialysis unit of hospitals and the dialysis referral center after explaining the objectives of the study. If a patient was unable to fill out the questionnaire or needed help due to a low level of education or illness, the researcher read the questions one by one to the patient and completed the questionnaire based on the answers.

### Data analysis

To analyze the data, the answers to 12 questions were scored from one to four (1 = strongly disagree, 2 = disagree, 3 = agree and 4 = strongly agree). The reverse scoring method was used for 16 questions so that the score 4 was used for strongly disagree and score 1 for strongly agree. The data were analyzed in SPSS version 22. A median statistical test was used to compare the median of variables (readiness and its items) with the average value of 2.5. Mann–Whitney test was used to examine the relationship between readiness score and demographic characteristics of the participants (gender, marital status, place of residence). The relationship between readiness score and age and education level of the participants was also examined using Kruskal–Wallis statistical test.

### Ethical considerations

The Research Ethics Committee of Kerman University of Medical Sciences (Ethics ID IR.KMU.REC.1397292) approved this study. In addition, participation in the study was voluntary and a questionnaire was provided to patients if they were willing to participate. An informed consent was obtained before filling the questionnaire. All methods were carried out in accordance with relevant guidelines and regulations defined by the ethics committee.

## Results

The total number of patients that were admitted to dialysis centers during the study period was 263 patients, all of whom were invited to study and completed the questionnaires (100% participation rate). The reason for the high participation of patients was the explanation of the research objectives by the researchers before distributing the questionnaires and inviting patients to participate by healthcare staff while waiting to receive services. Demographic information of the participants is shown in Table 1. According to the results, most of the patients were male, married, over 60 years old, had an education degree below high school diploma, and lived in the city (Table 1).

**Table 1:** Demographic information of patients with chronic renal failure in South Khorasan province

Variables	No	Percentage
*Sex*		
Male	153	58.2
Female	110	41.8
*Marital status*		
Single	26	9.9
Married	237	90.1
*Education*		
Illiterate	90	34.2
Lower than the high school diploma	104	39.5
High school diploma	41	15.6
Academic	28	10.6
*Place of residence*		
Urban	201	76.4
Rural	62	23.6
*Age*		
Lower than 30	19	7.2
59–30	107	40.7
Higher than 60	137	52.1
*Sum*	263	100

The median of the factors related to the patients’ readiness to use health information technology are shown in Table 2. The mean scores of all questions related to the factor "health information need", were between one and two. In this factor, the lowest mean score (1.42) was assigned to the item “If I went on the internet, I would use it to look up information about herbals and/or supplements". In the factor of "desire to receive information", patients gave the highest score to the item “People today want to know too much about their health" (3.43). The highest disagreement was with the item “I am concerned about what I might find if I look up health issues on the internet" with a mean score of 1.55. In the factor "computer/internet experience and expertise” the mean scores of all items were lower than 2.

**Table 2:** The median of the factors affecting the readiness of chronic kidney patients

Factors	Items related to each factor	Completely disagree No (%)	Disagree No (%)	Agree No (%)	Completely agree No (%)	Median (1st quartile–third quartile)
Health information need	If I went on the internet, I would use it to look up things so that I wouldn’t worry about them anymore	(8/73)194	(1/9)24	(2/12)32	(9/4)13	4(4–4)
	If I went on the internet, I would use it to look up information about herbals and/or supplements	(7/78)207	(2/7)19	(6/7)20	(5/6)17	4(3–4)
	If I went on the internet I would use it to look up symptoms	(4/73)193	(1/6)16	(2/12)32	(4/8)22	2(1–3)
	If I went on the internet, I would use it to search for information about my health	(1/74)195	(2/4)11	(11)29	(6/10)28	4(3–4)
	If I went on the internet, I would use the internet to find information about medications	(1/74)195	(9/4)13	(11)29	(9/9)26	1(1–3)
Desire to receive information	People today want to know too much about their health	(8/6)18	(6/4)12	(4/27)72	(2/61)161	1(1–2)
	Regarding my health, I agree with the statement “No news is good news”	(1/33)87	(9/20)55	(7/21)57	(3/24)64	3(2–4)
	I am concerned about what I might find if I look up health issues on the internet	(5/63)167	(9/20)55	(2/12)32	(4/3)9	1(1–2)
Computer/internet experience and expertise	If I went on the computer, I would be able to figure out most computer problems that I might run into	(8/79)210	(6/7)20	(3/10)27	(3/2)6	3(1–4)
	If I went on the computer, I would have access to the internet	(8/79)210	(2/4)11	(7/8)23	(2/7)19	1(1–1)
	If I went on the internet, I would find using the internet to be easy	(4/73)193	(8/3)10	(8/11)31	(11)29	1(1–2)
	If I went on the internet, I would find using email to be easy	(7/81)215	(7/8)23	(7/5)15	(8/3)10	1(1–2)
Computer/internet anxiety	If I went on the computer, I would find using it to befrustrating	(6/77)204	(6/10)28	(1/9)24	(7/2)7	1(1–3)
	If I went on the internet, I would get frustrated with the amount of information I found about health on the internet	(8/76)202	(16)42	(5/6)17	(8/0)2	1(1–1)
	If I went on the internet, I would find searching for information on the internet would be stressful	(76)200	(1/14)37	(4/8)22	(5/1)4	1(1–1)
	If I went on the internet, I would find sortingthrough information on the internet to be too time-consuming	(2/77)203	(1/14)37	(5/6)17	(3/2)6	1(1–1)
Preferred mode of interaction	I trust the internet as a source of health information	(2/58)153	(8/14)39	(8/19)52	(2/7)19	1(1–2)
	Looking up health concerns on the internet is more convenient for me than contacting a doctor’s office	(6/69)183	(16)42	(1/9)24	(3/5)14	1(1–1)
	I prefer calling my doctor’s office to emailing them	(5/36)96	(6/4)12	(5/20)54	(4/38)101	1(1–2)
	I send an email to my doctor because it's easier for me than visiting the office	(2/85)224	(3/10)27	(9/1)5	(7/2)7	1(1–2)
	Looking up information online about medications is easier than asking my doctor	(5/74)196	(3/10)27	(4/11)30	(8/3)10	1(1–1)
Relationship with Doctor	I let my doctor handle the details of my health	(9/1)5	(0)0	(8/14)39	(3/83)219	1(1–1)
	Doctors are my most trusted source of health information	(7/2)7	(3/2)6	(6/23)62	(5/71)188	1(1–1)
	When I have a health concern, my first step is to contact my doctor’s office	(3/24)64	(5/6)17	(27)71	(2/42)111	1(1–2)
Cell phone expertise	I go online using my cell phone	(7/70)186	(5/6)17	(4/8)22	(1/14)37	1(1–1)
	I send text messages to people using my mobile phone almost every day	(8/54)144	(11)29	(3/21)56	(9/12)34	1(1–2)
Internet privacy concerns	If I went on the internet, I would be very concerned about giving any personal information	(2/72)190	(6/10)28	(11)29	(1/6)16	1(1–2)
	If I went on the internet, I would be concerned it would lead to invasions of my privacy	(5/71)188	(5/6)17	(4/14)38	(6/7)20	1(1–2)

The means of all items in the "computer/ internet anxiety” factor were lower than 2. In the "preferred mode of interaction" factor, the item "I send an email to my doctor because it's easier for me than visiting the office” received the lowest score, with a mean of 1.22 (Table 2).

In determining the relationship of patients with their doctors, the lowest mean score (2.87) was assigned to the item "when I have a health concern, I call my doctor's office first", the item "I go online using my cell phone" had the lowest mean score (1.73) in the “cell phone expertise” factor. In identifying internet privacy concerns of chronic kidney patients, both items of concern about providing personal information and invading their privacy gained mean scores lower than 2 (Table 2).

The results showed that the mean scores of the factors “health information need”, “computer/internet experience and expertise”, “preferred mode of interaction”, “relationship with doctor”, and “cell phone expertise” were significantly lower than the average. The mean scores of “computer/internet anxiety” and “internet privacy concerns” were above average (*p* < 0.001) and the “desire to receive information” factor was moderate (*p* = 0.35) (Table 3).

**Table 3:** Median of readiness factors in chronic kidney patients

Variable	Mean ± standard deviation	(1st quartile–third quartile) median	Mann–Whitney test result
Health information need	89/0 ± 2/1	(2–1)1	76/12- = Z 001/0 > P
Computer/internet experience and expertise	78/0 ± 43/1	(75/1–1)1	32/13- = Z 001/0 > P
Computer/internet anxiety	57/0 ± 66/3	(4–25/3)4	19/14- = Z 001/0 > P
Preferred mode of interaction	55/0 ± 66/1	(2–20/1)6/1	29/13- = Z 001/0 > P
Relationship with Doctor	54/0 ± 57/1	(2–1)33/1	66/13- = Z 001/0 > P
Cell phone expertise	20/1 ± 83/1	(5/2–1)1	24/9- = Z 001/0 > P
Internet privacy concerns	91/0 ± 45/3	(4–3)4	16/12- = Z 001/0 > P
Desire to receive information	56/0 ± 55/2	(3–2)67/2	94/0- = Z 35/0 = P

Table 4 presents the differences of readiness in chronic kidney patients based on their demographic information. The results showed that there was a significant difference between the mean scores of men and women in all factors except the “desire to receive health information” factor. Men gave higher scores to all factors except to “computer/internet anxiety” and “internet privacy concerns” (*p* < 0.05). There was a significant association between participants’ age and all factors except the “preferred mode of interaction” with a physician. Except for three factors of “computer/internet anxiety”, the “desire to receive information” and “internet privacy concerns” the scores given by people under 30 years of age to other factors were higher. People over 60 years of age gave higher scores to these factors (*p* < 0.05). In all factors except the “preferred mode of interaction”, there was a significant difference between the scores of individuals based on their education. People with an academic degree gave higher scores to all factors except to the “computer/internet anxiety”, “desire to receive information” and “internet privacy concerns” (*p* < 0.05). There was a difference between the scores of single and married people in all factors except in the “preferred mode of interaction” with a doctor. Except in “computer/internet anxiety”, the “desire to receive information” and “internet privacy concerns” scores of singles were higher (*p* < 0.05). The results showed that in all factors except the “preferred mode of interaction”, and the “desire to receive information”, there was a difference between the scores of individuals. In all factors except “computer/internet anxiety”, and “internet privacy concerns” the scores given by urban residents were higher (*p* < 0.05).

**Table 4:** Differences of readiness in chronic kidney patients based on their demographic information

Variable	Sex: median (1st quartile–third quartile)	Mann–Whitney-test result	Marital status median (1st quartile–third quartile)	Mann–Whitney-test result	Education median (1st quartile–third quartile)	Mann–Whitney-test result	Age median (1st quartile–third quartile)	Mann–Whitney-test result
Health information need	Men 1 (1–2.60) Women1(1–1)	Z = -3.17 *P* = 0.002	Single:2.4(1–3.2) Married:1(1–1.4)	Z = 4.73 *P* < 0.001	Illiterate:1(1–1) Lower than the high school diploma:1(1–1.4) High school diploma:2.4(1–3) Academic:2.8(2.1–3.3)	Χ^2^ = 92.62 *P* < 0.001	Lower than 30:2.6(1–3.2) 59–30:1(1–2.6) Higher than 60:1(1–1)	Χ^2^ = 40.38 *P* < 0.001
Computer/internet experience and expertise	Men 1 (1–2) Women1(4–4)	Z = -3.26 *P* = 0.001	Single:2(1–3.5) Married:1(1–1.5)	Z = 4.11 *P* < 0.001	Illiterate:1(1–1) Lower than the high school diploma: 1(1–1) High school diploma:1.75(1–2.5) Academic2.63(1.75–3.25)	Χ^2^ = 100.77 *P* < 0.001	Lower than 30:2.25(1–3.5) 59–30:1(1–2) Higher than 60:1(1–1)	Χ^2^ = 42.25 *P* < 0.001
Computer/internet anxiety	Men 1 (3.25–4) Women1(4–4)	Z = -3.49 *P* = 0.001	Single:3.25(2.75–4) Married:4(3.5–4)	Z = 4.02 *P* < 0.001	Illiterate:4(4–4) Lower than the high school diploma:4(3.5–4) High school diploma:3.5(2.75–4) Academic: 3(2.75–3.25)	Χ^2^ = 85.43 *P* < 0.001	Lower than 30:3.25(2.75–4) 59–30:4(3–4) Higher than 60:4(4–4)	Χ^2^ = 38.11 *P* < 0.001
Preferred mode of interaction	Men 1 (1–2) Women1(1–2)	Z = -3.17 *P* = 0.002	Single:2.2(1.6–2.8) Married:1.6(1.2–1.8)	Z = 4.39 *P* < 0.001	Illiterate:1.5(1.2–1.6) Lower than the high school diploma:1.6(1.2–1.8) High school diploma:2(1.6–2.4) Academic: 2.4(1.7–2.8)	Χ^2^ = 59.78 *P* < 0.001	Lower than 30:2.2(1.6–2.8) 59–30:1.6(1.4–2.2) Higher than 60:1.6(1.2–1.6)	Χ^2^ = 29.38 *P* < 0.001
Relationship with Doctor	Men 1 (1–2) Women1(1–2)	Z = -2.50 *P* = 0.012	Single:1(1–1.67) Married:1.33(1–2)	Z = 0.79 *P* = 0.43	Illiterate:1.33(1–2) Lower than the high school diploma:1.33(1–2) High school diploma:1.67(1–2) Academic: 1.33(1–2.17)	Χ^2^ = 0.06 *P* = 0.99	Lower than 30:1.33(1–1.67) 59–30:1.67(1–2) Higher than 60:1.33(1–2)	Χ^2^ = 1.58 *P* = 0.45
Cell phone expertise	Men 1 (4–4) Women1(1–3)	Z = -0.50 *P* = 0.0.62	Single:3(2–4) Married:1(1–2)	Z = 4.51 *P* < 0.001	Illiterate:1(1–1) Lower than the high school diploma:2.4(1–3) High school diploma:3(2–3.5) Academic: 3(2–3.75)	Χ^2^ = 99.28 *P* < 0.001	Lower than 30:3(2–4) 59–30:2(1–3) Higher than 60:1(1–1.5)	Χ^2^ = 52.06 *P* < 0.001
Internet privacy concerns	Men 1 (2.5–4) Women1(1–1)	Z = -3.91 *P* = 0.001	Single:2.25(1.5–4) Married:4(3–4)	Z = 4.29 *P* < 0.001	Illiterate:4(4–4) Lower than the high school diploma:1.75(1–1.5) High school diploma:3(2–3.5) Academic: 3(2–3.75)	Χ^2^ = 2.33 *P* = 0.51	Lower than 30:2(1.5–4) 59–30:4(2.5–4) Higher than 60:4(4–4)	Χ^2^ = 36.59 *P* < 0.001
Desire to receive information	Men 1 (1–2.60) Women1(2–3)	Z = -1.60 *P* = 0.11	Single:2.33(2–2.67) Married:2.67(2–3)	Z = 2.86 *P* = 0.004	Illiterate:2.67(2.33–3) Lower than the high school diploma:3.5(2.75–4) High school diploma:2.67(2.33–3) Academic: 2.67(2–3)	Χ^2^ = 42.25 *P* < 0.001	Lower than 30:2.33(1.67–2.67) 59–30:2.33(2–3) Higher than 60:2.67(2–3)	Χ^2^ = 7.58 *P* = 0.02

## Discussion and conclusion

The results of this study on the readiness of chronic kidney patients to use health information technology showed that, since most chronic kidney patients are unfamiliar with how to search on the Internet, they do not trust its contents. Most of them are illiterate or unlearned, middle-aged with low income, and less likely to use technology-based resources such as the Internet. Studies [17–19] have shown that people often tend to seek health information. Therefore, it is necessary to remove barriers preventing patients to obtain information from electronic resources. Identifying the information needs and topics that patients are searching for on the Internet can help to develop the required content. The results of the present study showed that people are more likely to seek information about the symptoms of their disease and less likely to seek information about herbs or supplements. In this regard, the results of a study by Jafari Nodoushan et al. [18] also showed that most users did not use herbal medicines recommended on the Internet for their treatment.

In this study, most participants expressed that they wanted to know more about their health. Since patients with chronic renal failure undergo several treatments during their care process, to control the symptoms and complications, they should be acquainted with the causes of the disease, and various treatment methods [9]. In this regard, a study by Green et al. showed that people could play an effective role in preventing the progression of their disease by increasing their health-related information and self-management capacity [20]. Sometimes people express concern about the information they find on the Internet and do not trust it. In the study of Jafari Nodoushan, on thirds of people expressed that they had never trusted the knowledge and information obtained from the Internet without consulting a doctor, and often had not bought medications via the Internet [18].

In the present study, most people were unfamiliar with using email but liked to use the Internet. A review study by AlGhamdi et al. revealed that older people are usually not interested in the Internet, have not been properly trained to use it, and do not have the necessary self-confidence in this regard. The small font size of web pages and large amount of information on the Internet contribute to the lower use of this resource by older people. Therefore, they prefer to use traditional methods such as visiting a doctor [19]. Distrust of the Internet may also be a barrier to its use by the elderly. In this regard, the study of Ansari et al. [9] also showed that dialysis patients, due to insufficient reading literacy and their older age, obtain more information from physicians than the technology-based resources such as the Internet. The reason for this finding is that more than one third of the study population in study conducted by Ansari was illiterate.

Most patients found the use of computers boring. However, when they used the Internet, they were not disappointed about the amount of information they could find about their illness and had a positive outlook. In this regard, the results of the study of Lalehzarian et al. [21] showed that health information-seeking behavior is a psychological phenomenon that is affected by the individual characteristics of the patient. Various studies have shown that women feel less comfortable than men working with information technologies such as computers and the Internet and are more anxious when using these technologies [22, 23]. Studies conducted by Bigdeli et al. [24] and Feizabadi et al. [22] found that women have a positive view about health information on the Internet and there is a significant relationship between using the Internet and their positive view. Both study conducted among employed women this can affect their finding regarding the Internet use. Users' positive attitude towards using the Internet often leads to using the Internet to search for health information.

In this study, most patients preferred to visit the office or make a phone call to their doctor than sending an email. They also would trust their physicians and allow them to take care of their health conditions. A study by Santana et al. [15] found that individuals contact their physician, or other health care professionals, via email and relevant websites to request or review prescriptions, make appointments or ask specific health questions. Wallwiener et al. [16] by reviewing 71 articles, introduced electronic messages as another channel of communication between patients and their physicians and acknowledged their satisfaction with these services. In general, the results of many studies have shown that patients use the Internet to consult and communicate with a physician to obtain health information [15–17, 25]. Therefore, patients seek to use these services due to ease of use, access at any time and place, and their cost-effectiveness.

In the present study, patients mostly use mobile phones to send text messages to people every day and less use them to search on the Internet. The results of a study by Seto et al. showed that heart failure patients can take positive behaviors to change their lifestyle using mobile phones, and these facilities improve the clinical management of patients, self-care, and clinical feedback, and reduce clinical visits and hospitalizations by providing physiological information through alerts [26]. Therefore, mobile technology can be used in planning activities and clinical decisions, and managing signs, symptoms, and patient conditions [27].

Most patients in this study were concerned about invasion of their privacy if they use the Internet. Insufficient skills to identify trusted websites are among the problems that people face when searching and using the Internet to access online health information [28]. The results of some studies [28–30] have shown that the use of health information technology has an important role in meeting patient information needs and now the number of users who use these services is increasing. In many cases, it is the primary resource of health-related information. Nevertheless, there are still people who have insufficient knowledge about using the Internet and cannot use these services as health information resources. Insufficient knowledge of users in addition to the lack of trustworthy websites containing health information lead to the vulnerability of patients against unauthorized disclosure of their information.

In this study, men and women were equally inclined to receive information about their health and there was a significant difference between them in other factors determining the readiness of chronic kidney patients. Koopman et al. [13] showed that because of the role of the women in the family, they use the Internet and information technology more. Koopman’s study was conducted among patients with chronic conditions from family medicine clinics. In the present study, single and married people had similar opinions about the mode of interaction with their physicians and there was a significant difference between them in other factors that determine the readiness of chronic kidney patients. Married people were more anxious about using the computer, more interested in knowing about their health, and more concerned about the security and confidentiality of their information on the Internet. Among postgraduate students, single people have more time and more curiosity to use the Internet, so their readiness to use information technology is higher than other people [31]. In this study, people over the age of 60 were more anxious about computer use, more interested in knowing about their health, and more concerned about the security and confidentiality of their information on the Internet. In this regard, Powell et al. have shown that often women under the age of 45 search for online health information [32]. The results of other studies [1, 33, 34] have also reported a direct relationship between age and seeking health information of patients. With increasing age, the rate of active search for information in patients decreases. Since in older adults the individual and physical abilities for doing daily activities including the search for health information decreases, family members should also be involved in the searching and providing information to elder patients [21]. In the present study, a significant relationship was observed between the desire to receive health information and the education level of individuals and their history of specific diseases. In this regard, studies have shown [35, 36] people with a lower education level and those who have experienced a history of a particular disease in their lives need more health information. To encourage patients to use IT-based interventions and increase their readiness; training users, providing low-cost services and improving trust of users are possible solutions [37]. Moreover, improving patient confidentiality and security of the data play role in accepting the IT-based intervention.

The present study had three limitations. First, most patients were unable to fill out the questionnaire personally. To solve this problem, a trained researcher read the questions for respondents and filled out the questionnaire based on their responses. Second, since this study was performed on dialysis patients in one province, the results should be used with caution to patients in other regions. However, due to the multiplicity of centers and the high number of patients, the generalizability of the results might not be affected much. Third, the validity and reliability of the questionnaire may not have enough strength as patients did not invited to participate in the face validity and the reliability was assessed with the limited number of the participants.

## Conclusion

The results of the present study showed that the level of readiness of chronic kidney patients receiving hemodialysis to use health information technology is low. Since most patients in this study wanted to receive information about their disease, it is suggested to perform required interventions to improve the readiness of people to use health information technology. Healthcare authorities and policymakers should plan to invest more in providing electronic health solutions according to the level of patients’ readiness and capability by developing information and communication technology infrastructures.

## Data Availability

Data collected in this research can be obtained from the corresponding author if required (l.ahmadian@kmu.ac.ir).
